# Two-stage fluid pathways generated by volume expansion reactions: insights from the replacement of pyrite by chalcopyrite

**DOI:** 10.1038/s41598-020-76813-9

**Published:** 2020-11-17

**Authors:** Yang Zhang, Yuanfeng Cai, Yang Qu, Qin Wang, Lixin Gu, Gaojun Li

**Affiliations:** 1 State Key Laboratory for Mineral Deposits Research, School of Earth Sciences and Engineering, Nanjing University, 163 Xianlin Avenue, Nanjing, 210023 China; 2 Electron Microscopy Laboratory, Institute of Geology and Geophysics, Chinese Academy of Sciences, Beijing, 100029 China; 3 MOE Key Laboratory of Surficial Geochemistry, School of Earth Sciences and Engineering, Nanjing University, 163 Xianlin Avenue, Nanjing, 210023 China

**Keywords:** Solid Earth sciences, Geochemistry, Geophysics, Mineralogy

## Abstract

Volume expansion reactions involved in mineral–fluid interactions are linked to a number of geological processes, including silicate weathering, retrograde metamorphism, and mineralization. However, the effect of volume expansion on replacement reactions remains unclear. Here, we demonstrate that reactions associated with volume expansion during the replacement of pyrite by chalcopyrite involve two competing processes. The reaction is initially augmented because of the development of reaction-induced fractures in the pyrite. However, these fractures are subsequently filled by compacted products, which ultimately disrupts the contact and interaction between bulk fluids and the pristine pyrite surface. These competing processes indicate that replacement reactions are both augmented and inhibited by volume expansion reactions during pyrite replacement.

## Introduction

Volume expansion reactions have a significant impact on the rate of fluid–mineral interactions that occur in a number of settings, including retrograde metamorphism during exhumation of high-pressure and ultrahigh-pressure metamorphic rocks^1–3^, chemical weathering of silicate minerals^4,5^, peridotite serpentinization^6,7^, and hydrothermal mineralization^8–10^.

Previous studies have focused on the generation and propagation of fractures in rocks and minerals based on outcrop observations and textural analyses^1,5–7,11^. It has been proposed that self-stress in minerals associated with fluid–mineral interaction plays an important role in fracture propagation^3,7^. However, the effect of volume expansion on mineral replacement reactions remains unclear. Volume expansion can result in compaction of the reaction products on mineral surfaces, which inhibits the exchange of reactants between fluids and the surface of the parent mineral^12–15^. In contrast, the crystallization and growth of replacement minerals may trigger fracturing and promote reactions through the development of new reaction surfaces^5–7,11,16–18^. It is therefore critical to evaluate the influence of new reactive surface exposure on the rate of mineral replacement during volume expansion reactions.

Here, we investigate the replacement of pyrite by chalcopyrite under controlled conditions to study the rate of the replacement reaction in response to volume expansion, which is a type of volume-increasing reaction^19^. This replacement reaction was chosen for the following reasons: (1) pyrite crystals can be collected easily, and cut into specific shapes; (2) the rapid transformation at 200 ℃ allows observation on a laboratorial timescale; and (3) the simple compositions of pyrite and chalcopyrite eliminate the influence of chemical changes during hydrothermal experiments.

## Results

### Mineral textures

**Figure 1:**
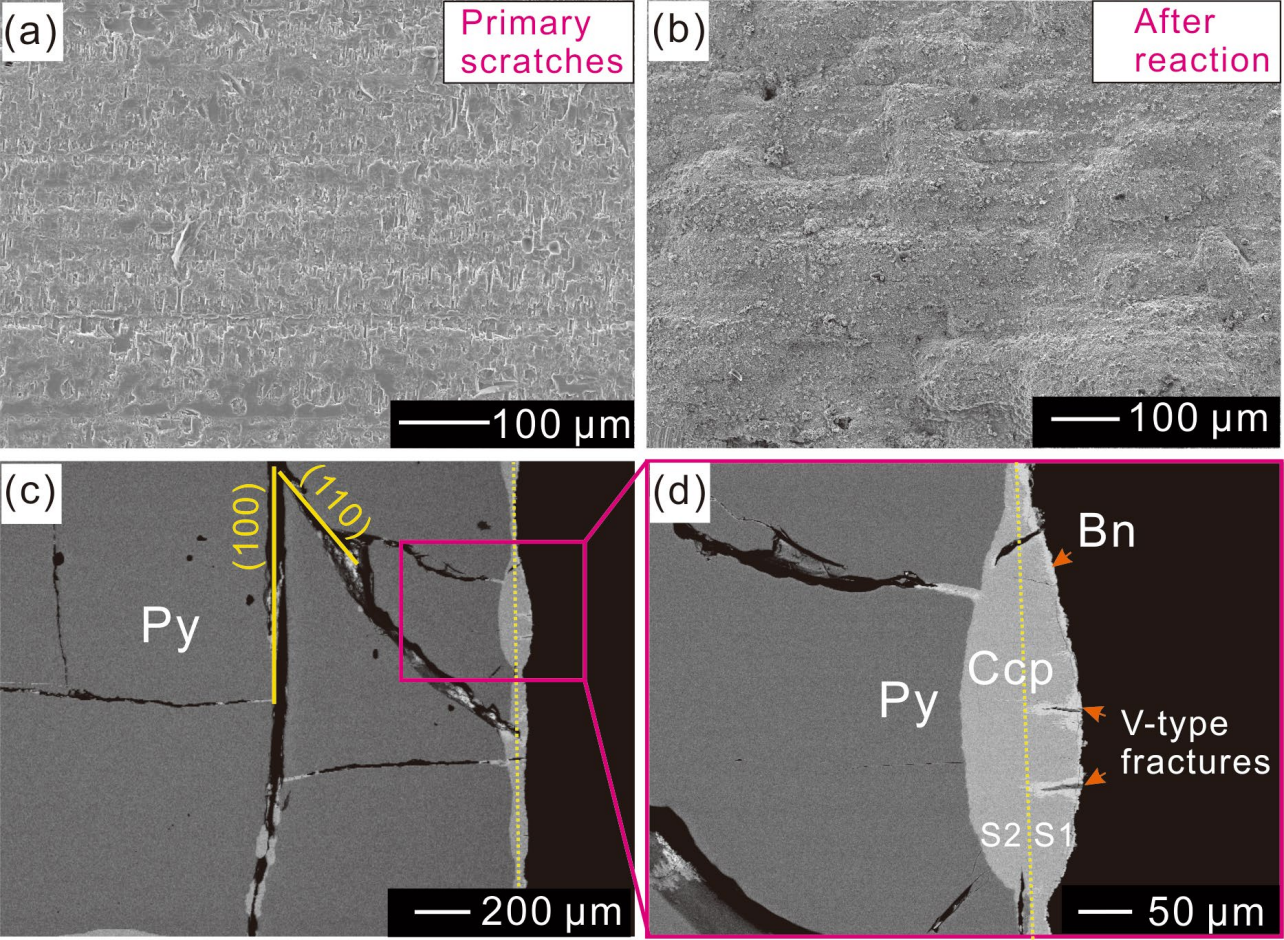
Scanning electron microscope images of reacted solids. (**a**,**b**) Secondary electron image showing surface scratches before (**a**) and after (**b**) the reactions. (**c**,**d**) Back-scattered electron images of solid cross-sections. (**c**) Partly reacted pyrite (Run No. 2). The dashed line shows the original pyrite surface, and the solid lines show fractures along the (100) and (110) planes. (**d**) Microscale volume increase. S1 (16,585 μm^2^) and S2 (19,701 μm^2^) are the increased and replaced areas, respectively. Py = pyrite, Ccp = chalcopyrite, Bn = bornite.

**Figure 2:**
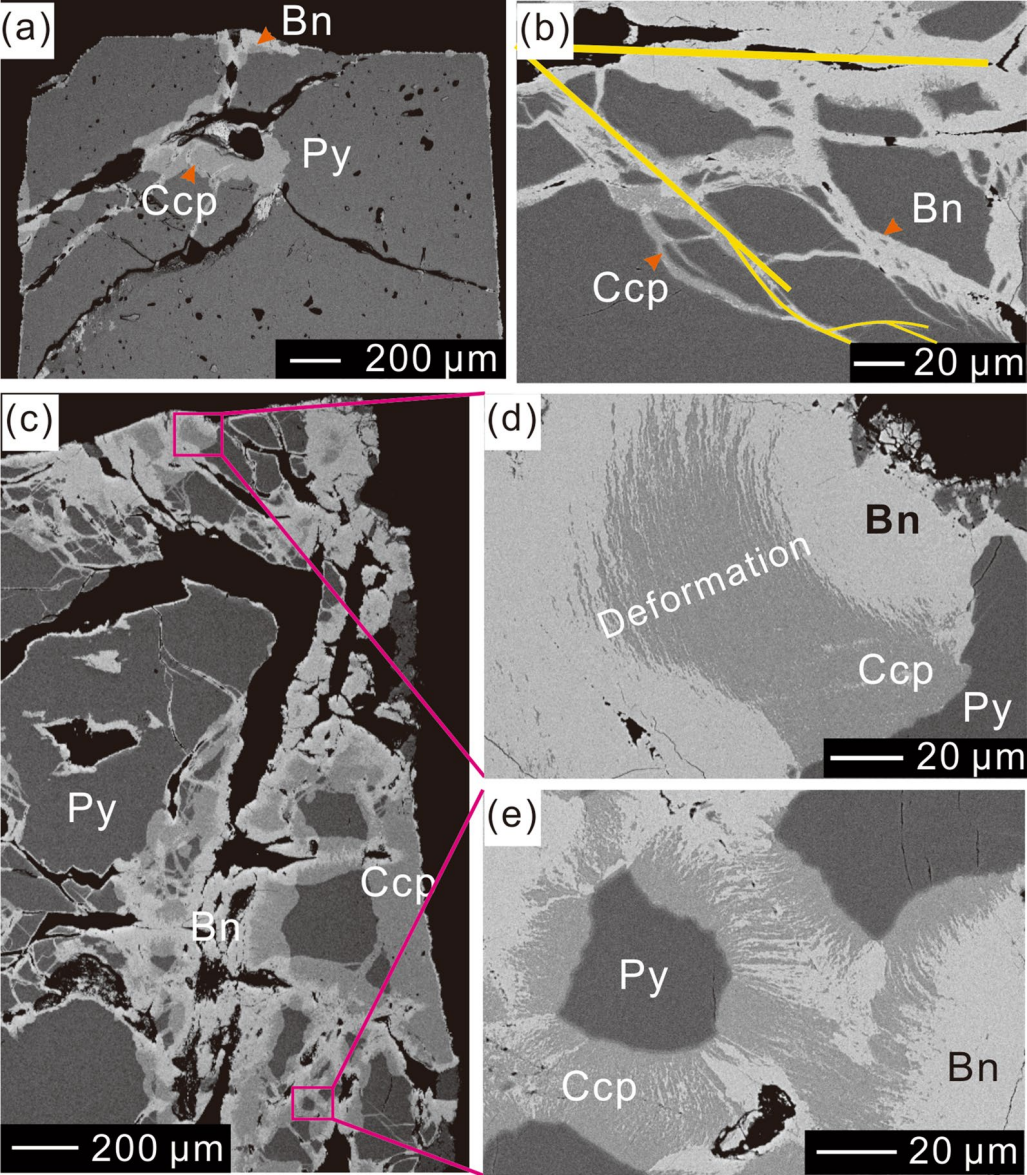
Scanning electron images showing fractures and deformation features. (**a**,**b**) Partly reacted pyrite showing different fracture patterns, including (**a**) radial fractures (Run No. 3), and (**b**) hierarchical fractures (Run No. 7). (**c**–**e**) Deformation textures of columnar chalcopyrite (Run No. 7). (**d**) and (**e**) Enlarged sections of (**c**).

**Figure 3:**
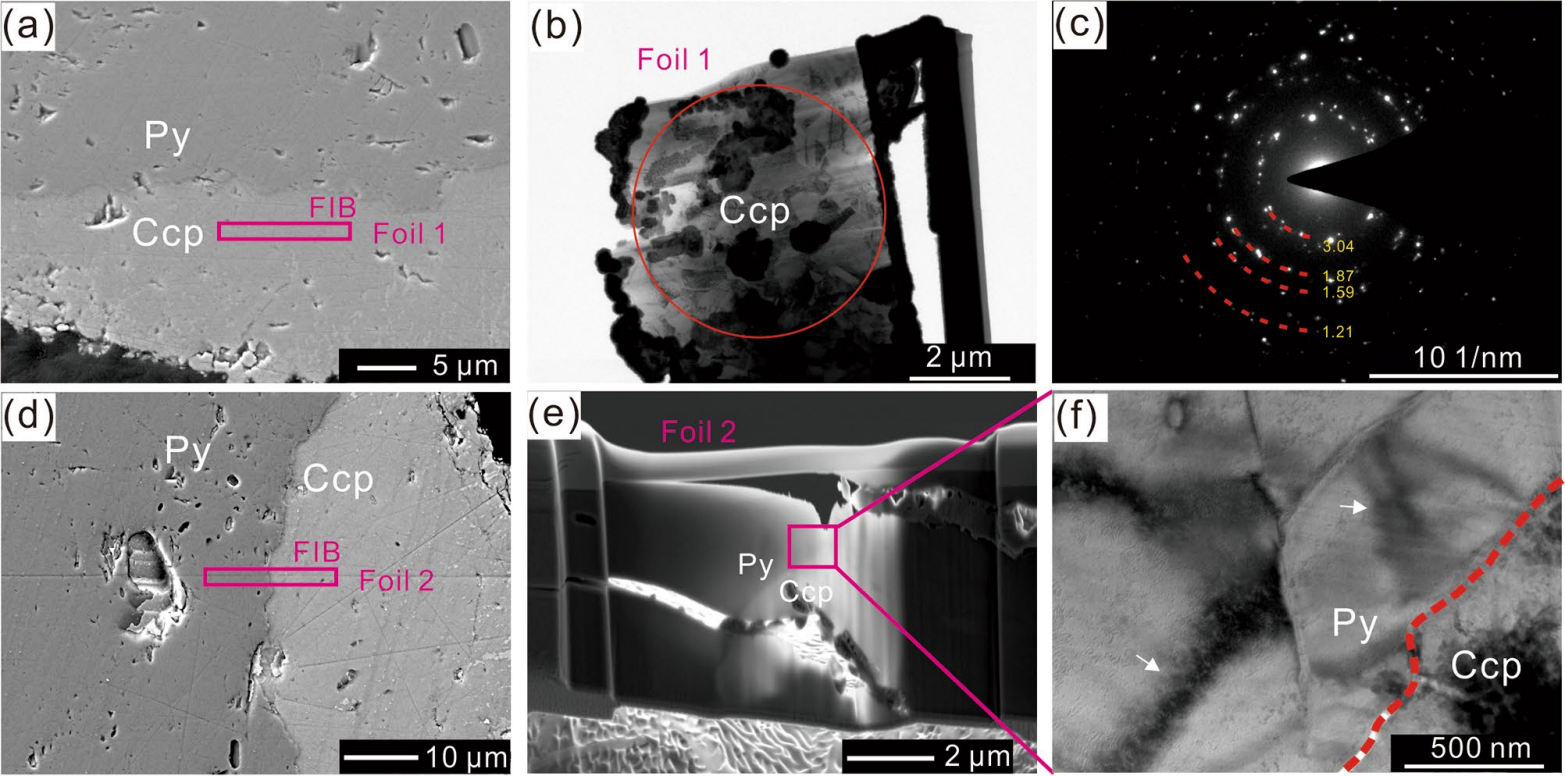
Focused ion beam–transmission electron microscope (FIB–TEM) images of pyrite and chalcopyrite. (**a**) and (**d**) are secondary electron images showing locations for TEM foils. (**b**) Bright-field TEM image of foil 1, showing chalcopyrite grains with different contrasts. The circle indicates the area in (**c**). (**c**) Selected area electron diffraction (SAED) pattern, showing that the chalcopyrite grains have different crystallographic orientations. (**e**) Secondary electron images for foil 2. (**f**) Bright-field TEM image, showing deformation features (marked with arrows) in pyrite.

Observations on solid surfaces demonstrate that pyrite surface scratches caused by sample preparation are well preserved on solid surface (Fig. 1a,b). Cross-section electron probe microanalysis (EPMA), micro-area and powder X-ray diffraction (μ-XRD) and Raman results indicate that chalcopyrite was the main product of the replacement reaction, with minor amounts of bornite and magnetite occurring on external mineral surfaces (Figs. 1c,d and 2a; Figs. S4–S6). The chalcopyrite has a columnar texture and no preferential crystallographic orientation, according to selected area electron diffraction (SAED) patterns (Fig. 3a–c). Pores are almost entirely absent in the chalcopyrite (Figs. 1d and 2d,e).

Replacement reactions were initiated in some areas of pyrite, with chalcopyrite developing on the surface of pyrite and forming mounds (Fig. 1c–d). Abundant unfilled fractures were observed before 21 days, and these fractures were generated during reactions (Fig. 1c), and only near-symmetrical chalcopyrite mounds were observed along fracture walls (Figs. 1c, 2a), with V-shaped fractures being observed on the solid surfaces of enlarged protruding mounds (Fig. 1d). An increase of 84% in volume in the replacement area was observed (Fig. 1d), which is in agreement with the theoretical molar volume increase (84%; 1 mol pyrite transition to 1 mol chalcopyrite) of the replaced phases. During the longer reaction time (Table 1, No. 7), deformation textures were observed in the resulting chalcopyrite (Fig. 2c–e), and transmission electron microscope images show deformation of pyrite near reaction surfaces (Fig. 3f).

**Table 1:** Measurement and calculation of changes in both volume and weight.

Run	Time (d)	L_b_ (mm)	V_b_ (mm^3^)	Wt_b_ (mg)	L_e_ (mm)	V_e_ (mm^3^)	Wt_e_ (mg)	ΔV (%)	ΔWt (%)
No. 1	5	2.00 * 2.06 * 2.16	8.772	39.6	2.06 * 2.10 * 2.12	9.189	40.7	4.757	2.778
		1.96 * 2.08 * 2.10			2.00 * 2.10 * 2.22				
		2.00 * 2.06 * 2.20			2.06 * 2.10 * 2.10				
		1.98 * 2.06 * 2.10			2.04 * 2.10 * 2.14				
No. 2	11	1.96 * 2.00 * 2.10	8.336	38.9	2.00 * 2.04 * 2.18	9.009	40.2	8.084	3.342
		1.98 * 2.00 * 2.10			2.04 * 2.08 * 2.20				
		1.98 * 2.04 * 2.10			2.04 * 2.08 * 2.16				
		1.98 * 1.98 * 2.12			2.02 * 2.02 * 2.12				
No. 3	14	2.00 * 2.06 * 2.10	8.747	38.7	2.10 * 2.12 * 2.16	9.616	40.2	9.936	3.876
		1.94 * 2.06 * 2.10			2.10 * 2.14 * 2.16				
		1.98 * 2.06 * 2.18			2.08 * 2.14 * 2.16				
		1.94 * 2.14 * 2.18			2.08 * 2.12 * 2.16				
No. 4	21	1.96 * 1.98 * 2.10	8.054	37.8	2.00 * 2.12 * 2.14	9.160	39.5	13.743	4.498
		1.94 * 1.98 * 2.04			2.00 * 2.10 * 2.16				
		1.96 * 2.00 * 2.06			2.00 * 2.16 * 2.16				
		1.98 * 1.98 * 2.08			2.02 * 2.12 * 2.14				
No. 5	34	2.00 * 2.04 * 2.06	8.384	40.9	2.14 * 2.14 * 2.20	10.790	43.4	28.686	6.112
		2.00 * 2.04 * 2.06			2.20 * 2.20 * 2.36				
		2.00 * 2.04 * 2.04			2.16 * 2.18 * 2.18				
		2.00 * 2.04 * 2.06			2.20 * 2.26 * 2.30				
No. 6	48	2.04 * 2.06 * 2.06	8.468	39.5	2.22 * 2.32 * 2.38	13.157	46.7	55.381	18.228
		2.00 * 2.04 * 2.06			2.34 * 2.42 * 2.44				
		2.00 * 2.04 * 2.06			2.28 * 2.32 * 2.50				
		2.00 * 2.04 * 2.06			2.28 * 2.40 * 2.44				
No. 7	63	2.00 * 2.00 * 2.02	8.120	39.7	2.18 * 2.50 * 2.56	13.583	49.2	67.281	23.929
		2.00 * 2.00 * 2.04			2.30 * 2.40 * 2.40				
		2.00 * 2.00 * 2.04			2.38 * 2.38 * 2.38				
		1.98 * 2.00 * 2.04			2.36 * 2.40 * 2.40				

L_b_ = edge length of pyrite before reaction. V_b_ = pyrite volume before reaction. W_tb_ = pyrite weight before reaction. L_e_ = edge length of solid after reaction. V_e_ = solid volume after reaction. W_te_ = solid weight after reaction. ΔV = 100 × (V_e_ − V_b_)/V_b_. ΔWt = 100 × (W_te_ − W_tb_)/W_tb_.

Abundant fractures were generated during replacement reactions. The fractures can be divided into three types: radial fractures (Fig. 2a), fractures along pyrite cleavage planes (Fig. 1c), and hierarchical fractures (Fig. 2b). During experiments with shorter durations, open fractures typically separated large pyrite grains into smaller domains (Figs. 1c and 2a), whereas fractures that formed over longer periods were filled by products (Fig. 2c).

### Solid volume (weight) change and quantification of fractured pyrite grain perimeters

**Figure 4:**
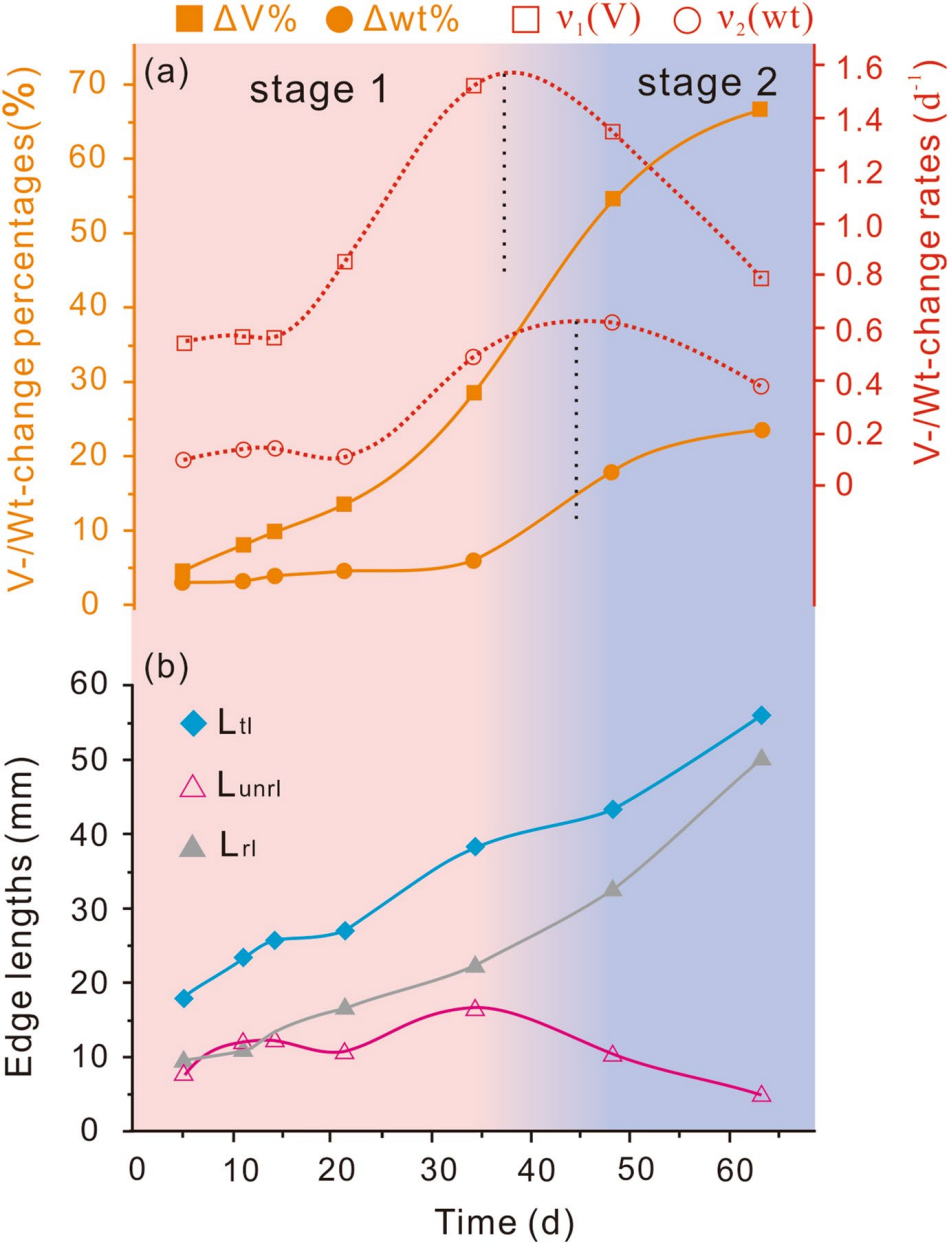
Changes in volume, weight, and pyrite edge lengths during the reaction. (**a**) Orange lines show the percentage changes in volume (ΔV%) and weight (ΔWt%) of solids, and dashed red lines are the change rates of ΔV% and ΔWt%; ΔV% = 100 × (V_e_ − V_b_)/V_b_, ΔWt% = 100 × (Wt_e_ − Wt_b_)/Wt_b_. V_b_ = pyrite volume before reaction; Wt_b_ = pyrite weight before reaction; V_e_ = solid volume after reaction; Wt_e_ = solid weight after reaction; v_1_ = change rate of ΔV%; v_2_ = change rate of ΔWt%. (**b**) Quantified lengths of grain boundaries. The blue line shows the total perimeter (L_tl_) of pyrite grains, the gray line shows the replaced length (L_rl_) of pyrite grains, and the red line shows the unreplaced length (L_unrl_).

Post-reaction solid volume and weight increased gradually with time from 5 to 63 days (Fig. 4a). The maximum increases (Table 1, No. 7) that were observed in the volume and weight were ~ 67% and ~ 24%, respectively. The change rates for both volume and weight indicate a two-stage process (Fig. 4a). In the first stage, the volume change rate increased from 0.6 d^−1^ after ~ 5 days to 1.6 d^−1^ after ~ 35 days, and the weight change rate increased from 0.1 d^−1^ after ~ 5 days to 0.6 d^−1^ after ~ 45 days (Fig. 4a). The peak change rate for volume occurred around day 35, whereas the peak change rate for weight occurred around day 45 (Fig. 4a). The Particles (Pores) and Cracks Analysis System (PCAS) calculations revealed a fold increase of unreplaced length between days 5 and 34 (Fig. 4b). In contrast, during the second stage (between days 45 and 63), the change rates for both volume and weight (Fig. 4a), as well as the unreplaced fracture lengths (Fig. 4b), decreased in line with the duration of the experiment.

## Discussion

A clear increase is observed in post-reaction solid volumes at both microscopic and macroscopic scales (Figs. 1c,d, 4a). The molar volume and relative solubility of the parent mineral and reaction product are important factors to consider when determining the volume increase involved in interface coupled dissolution–reprecipitation (ICDR) reactions^12,20^. The dissolution of pyrite and precipitation of chalcopyrite can be written as:

1 $${\text{FeS}}_{{2}} + {\text{ 4H}}^{ + } + {\text{ 2Cl}}^{ - } + {\text{ 2e}}^{-} = {\text{ FeCl}}_{{{2}({\text{aq}})}} + {\text{ 2H}}_{{2}} {\text{S}}_{{({\text{aq}})}}$$

2 $${\text{CuCl}}_{{2}}^{ - } + {\text{ FeCl}}_{{{2}({\text{aq}})}} + {\text{ 2H}}_{{2}} {\text{S}}_{{({\text{aq}})}} = {\text{ CuFeS}}_{{2}} + {\text{ e}}^{ - } + {\text{4Cl}}^{ - } + {\text{ 4H}}^{ + }$$

**Figure 5:**
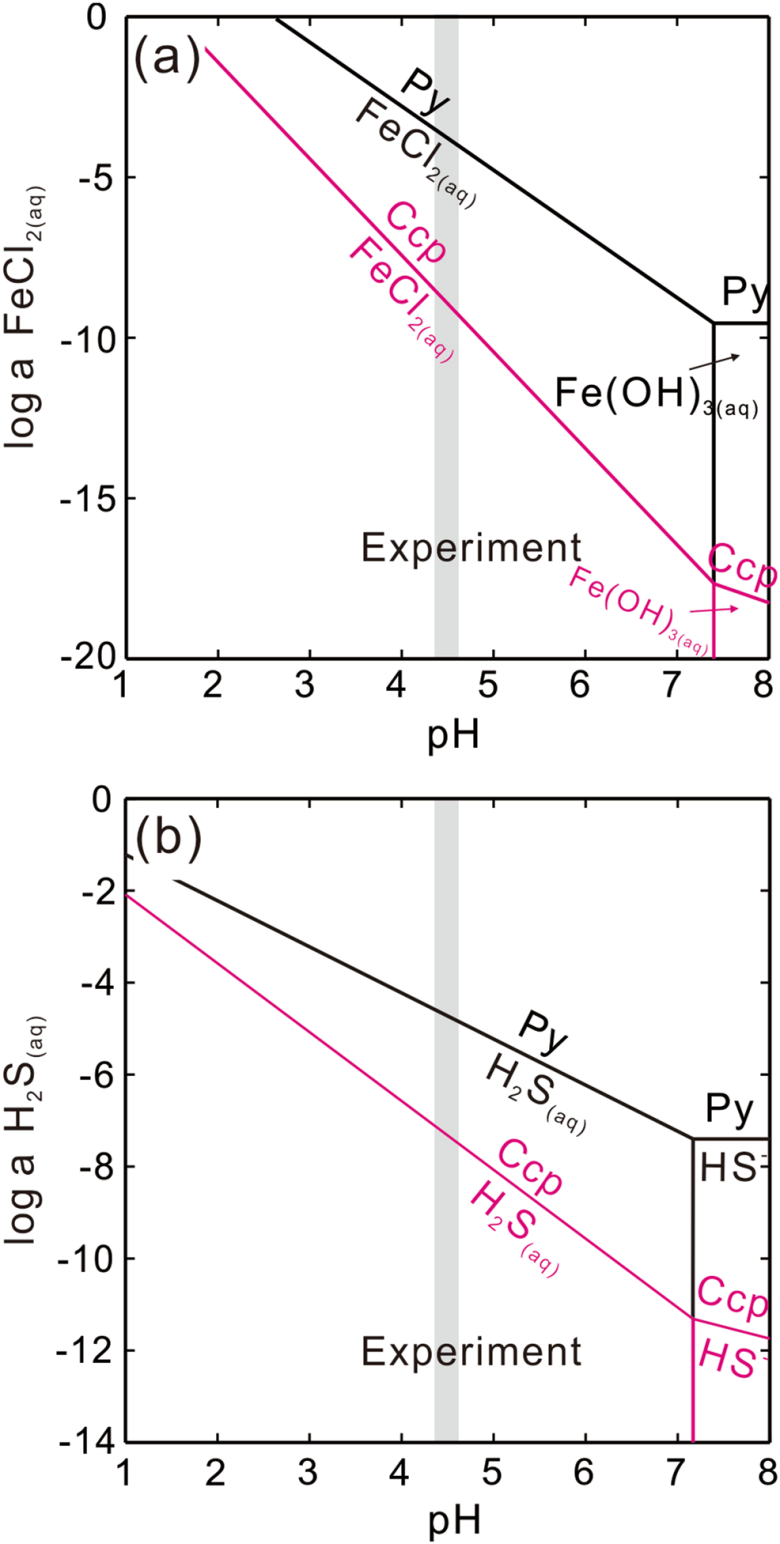
Solubility diagram of pyrite (black lines) and chalcopyrite (red lines) at 200 ℃, for (**a**) logaCl^−^  = 0, logaH_2_S =  − 3, aCuCl_2_^−^  = 0.2 M, and for (**b**) logaCl^−^  = 0, logaFeCl_2(aq)_ =  − 3, aCuCl_2_^−^  = 0.2 M. Diagrams were created using the Geochemist’s Workbench. The database used was thermo.dat with the association constant of Fe(II)–Cl species calculated from Testemale et al.^45^.

Area calculation from the cross-section (Fig. 1d) demonstrates that the actual volume change was in good agreement with the theoretical molar volume increase, suggesting that nearly all Fe and S that were dissolved during pyrite dissolution (Eq. 1) were precipitated as chalcopyrite (Eq. 2), which would generate an ~ 84% volume increase (Fig. 1d). However, the preservation of orientated surface scratches suggests a lack of obvious migration of Fe or S into the bulk solution and subsequent precipitation on the solid surface. This excludes the overgrowth mechanism, as any surface micro-textures would be destroyed^8^. Moreover, solubility diagrams indicate that the required concentration of FeCl_2(aq)_ or H_2_S_(aq)_ for chalcopyrite precipitation can be up to 3–5 orders of magnitude lower than that required for pyrite (Fig. 5a,b), suggesting that minor pyrite dissolution is sufficient for the supersaturation of chalcopyrite. Taken together, the factors described above support the dissolution of pyrite as the rate-limiting step in the reaction, with most of the Fe and S being precipitated near the reaction surface.

The nucleation and growth of chalcopyrite generate stress near reaction fronts. Using the standard free energy change of a chemical reaction, the crystallization pressure can be estimated by (e.g.^21^):

3 $$\Delta {\text{P }} = \, \Delta {\text{G }}/ \, \Delta {\text{Vs}}$$

where ΔVs is the actual solid volume change for per molar solid products, and can be considered the molar volume change in this study. ΔG can be calculated by the following chemical equation:

4 $$\begin{aligned} {\text{FeS}}_{{2}} + { 14 }/{\text{ 15CuCl}}_{{2}}^{ - } + { 8 }/{\text{ 15H}}_{{2}} {\text{O }} & = { 14 }/{\text{ 15CuFeS}}_{{2}} + { 14 }/{\text{ 15H}}^{ + } \hfill \\ & \quad + { 1 }/{\text{ 15FeCl}}_{{{2}({\text{aq}})}} + { 2 }/{\text{ 15HSO}}_{{4}}^{ - } + { 26 }/{\text{ 15Cl}}^{ - } \hfill \\ \end{aligned}$$

The calculated ΔP generated during crystallization of chalcopyrite can reach 700 MPa (200 ℃). Although stress can be released in several processes during mineral–fluid reactions^7,16^, the simple estimation of crystallization pressures demonstrates that the crystallization of reaction products can generate sufficient stress to induce fracturing or deformation (Figs. 1c, 2, 3), independent of any external stress (1.5 MPa, vapor saturated pressure at 200 ℃).

**Figure 6:**
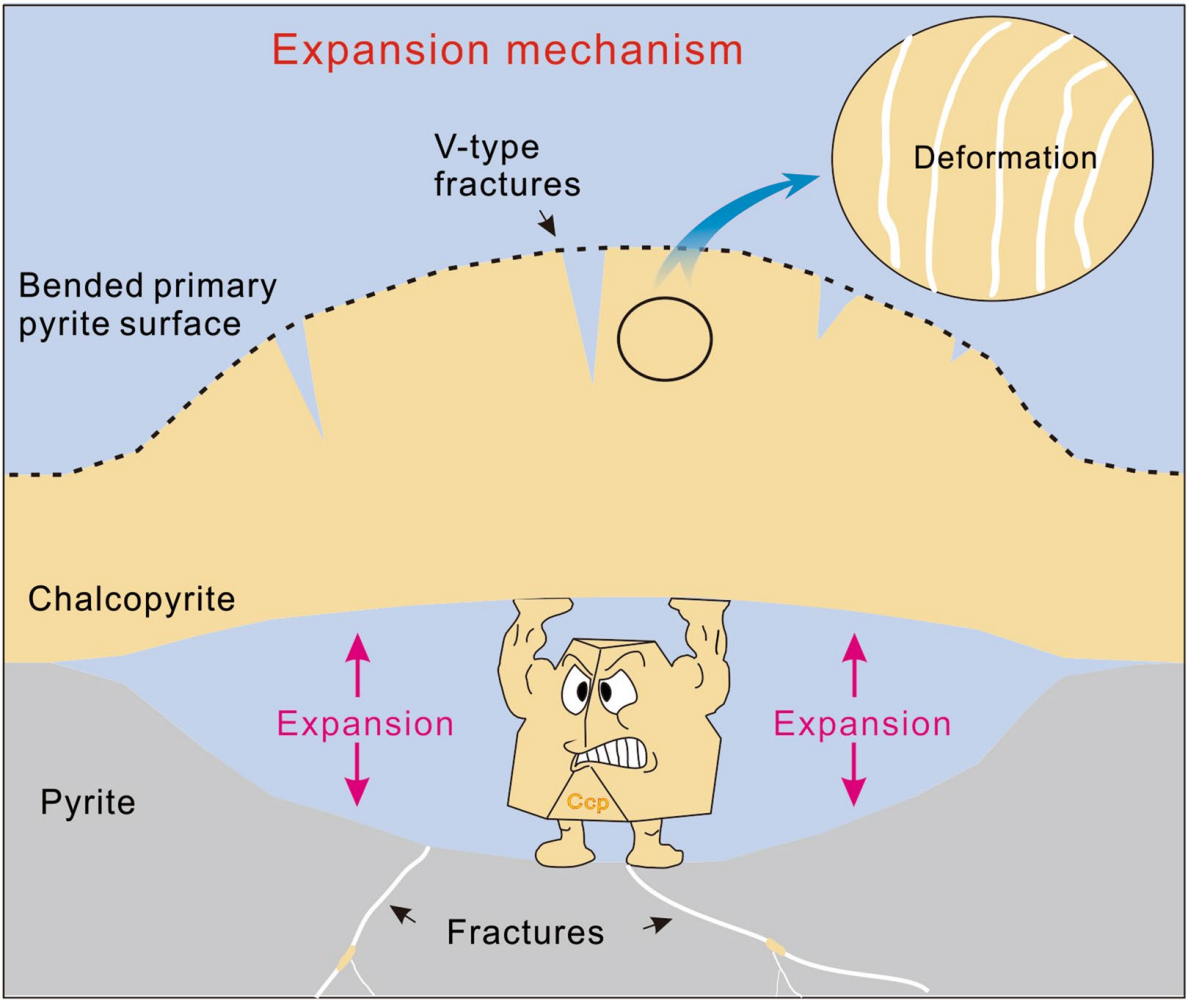
Diagrams illustrating expansion mechanism. The expansion mechanism is a dissolution-limited volume-increasing reaction. The crystallization and growth of chalcopyrite near reaction fronts pushes products outward, leading to an increase in solid volume, with simultaneous generation of fractures and deformation. Products are compacted with low permeability.

**Figure 7:**
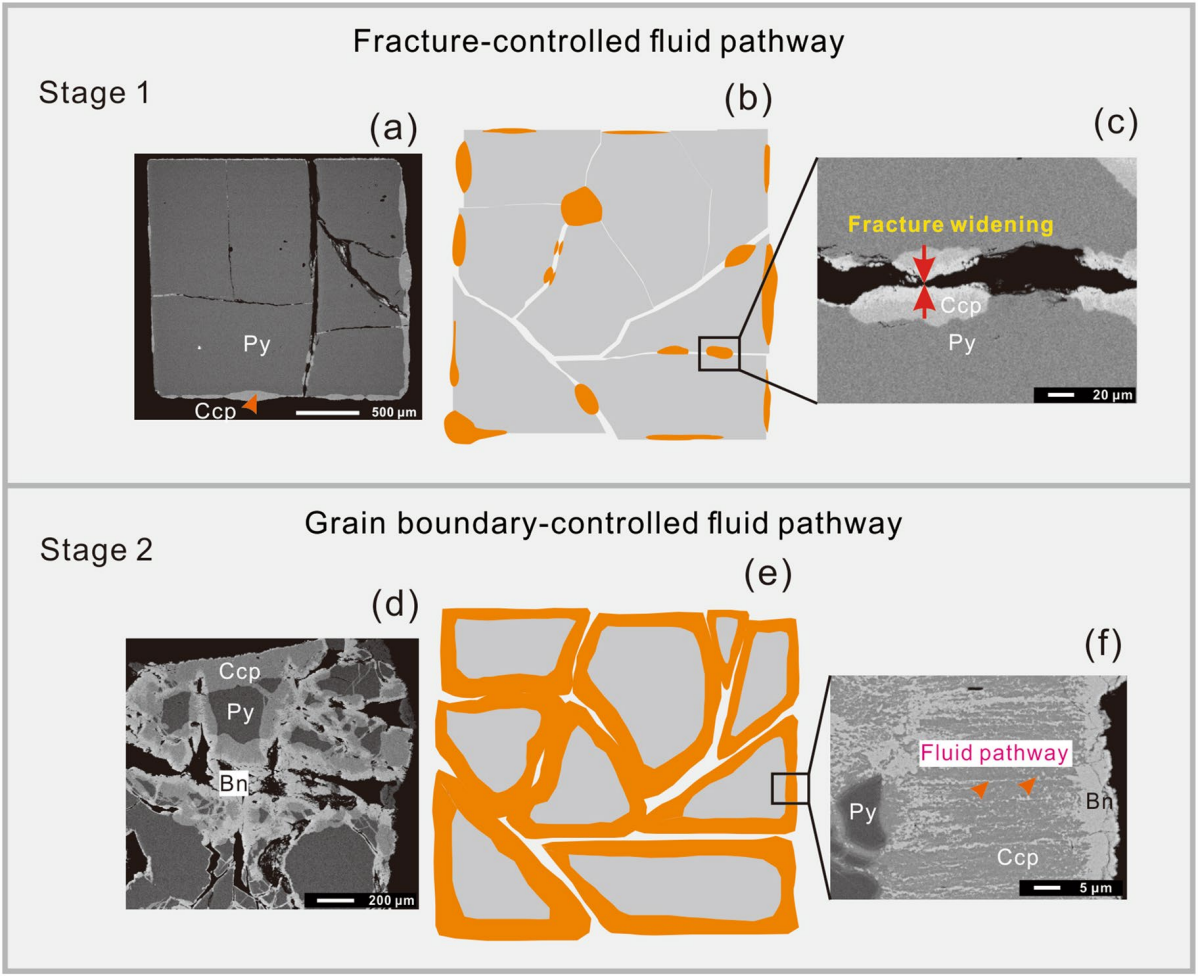
Two-stage fluid pathway processes. In stage 1, the fluid pathway is controlled by fracture generation. Abundant unfilled fractures expose more reactive surface; therefore, the reaction rate increases. In stage 2, the fluid pathway is controlled by grain boundaries. Abundant fractures are filled by compacted products, and the reaction rate is impeded.

This study demonstrates that the replacement of pyrite by chalcopyrite occurs via a volume expansion reaction. The heterogeneous nucleation of chalcopyrite and its growth near pyrite reaction fronts push the primary-formed products outwards, causing an increase in solid volume as well as deformation of the parent mineral and reaction products (Fig. 6). However, if a volume-increasing reaction is precipitation limited (Overgrowth mechanism), then the solutes cannot precipitate completely near reaction fronts (as illustrated in Figure 7 of reference 9)^9^. As a result, stress may not build up sufficiently for abundant fractures and deformation features to act as an expansion mechanism^9^.

Micro-textures have been used to reconstruct the deformation history of geographic areas in previous studies (e.g.^22,23^). However, the present study indicates that the crystallization and growth of reaction products can generate deformation textures during dissolution–precipitation reactions. Self-deformation textures have been reported in previous studies (e.g.^24–26^). As such, it is necessary to consider volume expansion when using micro-textures of minerals to reconstruct deformation histories of geographic areas.

Continuous reaction processes require free flow between the solution reservoir and the solid reactant. Fluid pathways, therefore, play a critical role in facilitating mineral–fluid reactions^27,28^. It is still not clear whether replacement reactions are promoted or inhibited during volume expansion reactions^29^. In this study, the two-stage processes described above (Fig. 7) can be attributed to the controlling influence of the reactive surface exposure, as both temperature and pressure remained constant during the reaction, and no more than 10% of Cu in solution was consumed (as calculated from the weight change before and after reactions).

Previous studies of silicate minerals have demonstrated that exposure of pristine surfaces can have a positive influence on the reaction process (e.g.^6,7,11,16,30–32^). In the first stage of our experiments, a large pyrite grain was cut into smaller pieces, exposing new reactive surfaces (Figs. 1c, 2a, and 7a–c). As reaction time increased, both the total length and unreplaced length increased (Fig. 4b), indicating that the continuous exposure of new pristine surfaces promoted the reaction.

A key requirement for the progression of replacement reactions is that the bulk solution can access the reaction surface^20,33^. However, products generated during volume expansion are usually compacted with low permeability^12,14^, which may inhibit the reaction^34^. As shown in Fig. 4, during the second stage, the change rates of both volume and weight did not increase in line with total pyrite length but decreased with the unreplaced length of pyrite grains. The change in reaction rate can be attributed to the low permeability of compacted reaction products (Figs. 1c–e and 7d–f), which hindered the reaction.

The two-stage fluid pathways observed imply that continuous exposure of pristine surfaces is the controlling factor in this volume expansion reaction. Between reaction runs Nos 5 and 6, the unreplaced lengths of pyrite grains began to decrease (Fig. 4b), suggesting that pyrite was rapidly covered by chalcopyrite. The textural features observed demonstrate that nucleation and growth of chalcopyrite not only generates fractures, but also causes deformations (Figs. 2d,e and 3f). These deformations can increase the reactivity of pristine surfaces^e.g.^^35^, upon which chalcopyrite develop more rapidly. These two competing processes (fracture generation and pristine surface coverage) cause the replacement reaction to eventually become a two-stage process. Of note, the peak change rate for weight lagged behind that of volume, indicating that the fractures were preferentially filled chalcopyrite over creating new pristine surface exposures.

The two-stage fluid pathway process implies that completion of volume expansion reactions, such as retrograde metamorphism of high-pressure and ultrahigh-pressure metamorphic rocks, and the transition from iron sulfides to copper-bearing sulfides in ore deposits, is difficult. For example, the primary fractures generated during volume expansion reactions, such as hydration of troctolite or weathering of dolerite, would increase the permeability of solids, which promotes solid–fluid reactions^1,5^. However, the replaced unbalanced textures of volume-expanding reactions are usually observed from thin sections^3,36^. The present study shows that the compacted products isolated the contact between fluids and pristine solid, resulting in the replaced unbalanced textures being more easily preserved over geological time scales (e.g.^37,38^). Therefore, unbalanced textures should be interpreted with caution regarding reflecting a short-term mineral–fluid reaction. Furthermore, our study implies that replacement reactions, such as silicate weathering, may also follow the two-stage model, in that original fractures are usually filled with secondary products (e.g.^21,39^). These products affect the contact between fluids and the reactive surface (e.g.^40–42^); therefore, the change in reaction rate resulting from different fluid pathways must be considered when comparing the reaction rate of silicate weathering in laboratory time scales with that in field studies.

## Materials and methods

Natural pyrite crystals (~ 1 cm in size) from Hunan Province, China, were used in this study. Purities of the pyrite crystals were checked by powder X-ray diffraction (XRD; Appendix Fig. S1) and field emission scanning electron microscopy (FE-SEM). To obtain consistent roughness on solid surfaces, pyrite crystals were first polished using silicon carbide sandpaper (P 1000), then cut into 2 mm cubes (Appendix Figs. S2, S3) using a low-speed diamond saw. The cubes were unbroken and contained no evident fractures (Appendix Fig. S2). All samples were cut along the (100) plane, and cubes were cleaned ultrasonically. The sides of the cubes were measured four times to calculate the average volume (Table 1). The cubes were then preserved in ethyl alcohol prior to the experiments.

Aqueous CuCl solution was used as the Cu source for pyrite reactions, and 1 M NaCl was added to prevent disproportionation of Cu^+^ to Cu^2+^ and Cu^0^ in aqueous solutions (e.g.^43,44^). The pH was buffered at 4.5 using acetate solution (0.1 M CH_3_COOH + 0.1 M CH_3_COONa + 1 M NaCl) and prepared with O_2_-free deionized water. All reagents were of analytical grade. Samples were prepared in a nitrogen atmosphere in an anaerobic glove box. A single pyrite cube, 150 mg CuCl, and 8 mL pH buffer solution were loaded into a 12 mL PTFE tube, which was then sealed in a stainless-steel bomb. Hydrothermal experiments were conducted in an electric oven at 200 °C with saturated vapor pressure (~ 1.5 MPa at 200 °C). The duration of each experiment varied from 5 to 63 days.

After the reactions, the PTFE-lined stainless-steel bombs were quenched in cold water for 15 min to stop the reaction. Cubes were extracted from the solutions, and carefully cleaned, air-dried, and weighed. The sides of the cubes were measured using Vernier Calipers (± 0.02 mm; Table 1) before and after the reactions. As the extent of the reaction is sensitive to changes in the solid volume and weight, rates of change in volume- and weight-percentages with time were used to determine the extent of the reaction.

The surface texture of the reacted cubes was first observed using a Carl Zeiss Supra 55 scanning electron microscope. The solids were mounted and polished gently to exposure inner textures for further observation. Mineralogical analysis of the reaction products was carried out using a Rigaku D/max Rapid II micro-diffractometer, and Rigaku 2DP software was used to analyze the data. A LabRAM HR800 Horiba Raman system was used to acquire the Raman spectra at microscale, using a 532.11 nm laser, and 600 groove mm^−1^ grating. Quantitative chemical analysis was performed using a JEOL 8100 electron microprobe running at 15 kV and 20 nA, with a defocused beam of 10 μm diameter. Data were corrected using standard ZAF correction procedures. Transmission electron microscopy (TEM) foil was prepared using a focused ion beam system on a Zeiss Auriga compact instrument at the Institute of Geology and Geophysics, Chinese Academy of Science. An FEI Tecnai F20 TEM instrument was used to analyze the texture and composition of foil samples.

A commercial Particles (Pores) and Cracks Analysis System (PCAS) was used to calculate the perimeters of fractured pyrite grains in the polished sections. Detailed descriptions of this software have been reported previously^45,46^. In brief, the SEM images were first transformed to binary images, then eroded into different small domains (pyrite grains). The total perimeters (L_tl_) of different domains were identified and calculated, as were unreplaced lengths (L_unrl_) of pyrite grains. Finally, the replaced lengths (L_rl_) of pyrite grains (L_rl_ = L_tl_ − L_unrl_) were calculated (Appendix Table S1).

## Supplementary information

Supplementary Information.
