# Supplementary material for: Two-stage fluid pathways generated by volume expansion reactions: insights from the replacement of pyrite by chalcopyrite
Source: Sci Rep. 2020 Nov 17;10:19993. doi: 10.1038/s41598-020-76813-9 (PMC7673132; doi:10.1038/s41598-020-76813-9)
Supplement: Supplementary file 1 — Supplementary Information. [file 41598_2020_76813_MOESM1_ESM.pdf]

SUPPLEMENTARY DATA

Two-stage fluid pathways generated by volume expansion reactions: Insights from the replacement of pyrite by chalcopyrite

Yang Zhang[1], Yuanfeng Cai[1*], Yang Qu[1], Qin Wang[1], Lixin Gu[2], and Gaojun Li[3]

[1]State Key Laboratory for Mineral Deposits Research, School of Earth Sciences and Engineering, Nanjing University, 163 Xianlin Avenue, Nanjing 210023, China

[2]Electron Microscopy Laboratory, Institute of Geology and Geophysics, Chinese Academy of Sciences, Beijing 100029, China

[3]MOE Key Laboratory of Surficial Geochemistry, School of Earth Sciences and Engineering, Nanjing University, 163 Xianlin Avenue, Nanjing 210023, China

**Corresponding author. E-mail: caiyf@nju.edu.cn*

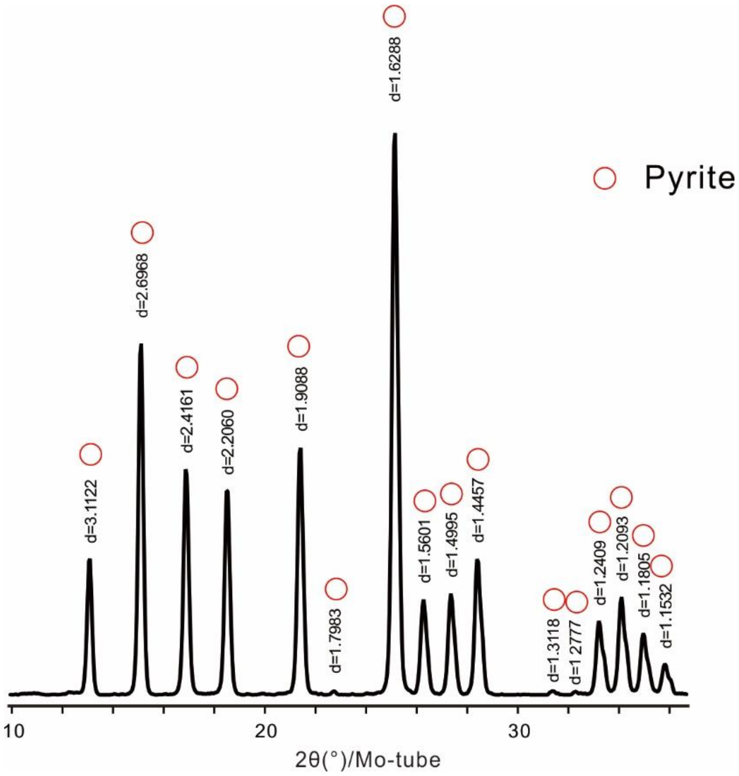

Fig. S1 X-ray diffraction pattern for the pyrite crystal used in this study, showing no phases other than pyrite.

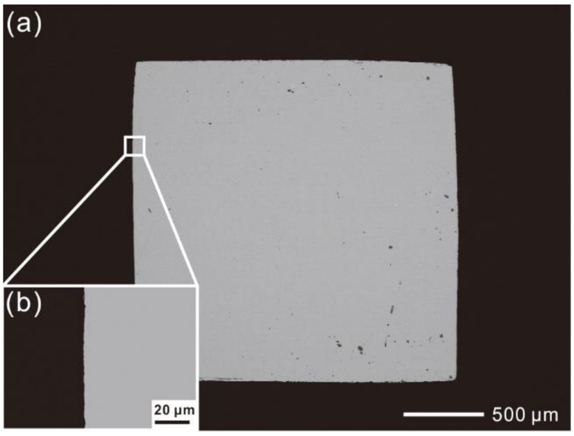

Fig. S2 Backscattered electron image of a pyrite cube cut by a low-speed diamond saw, showing that the incised pyrite was unbroken, with no primary fractures, and had straight boundaries. Some quartz and dolomite inclusions are visible.

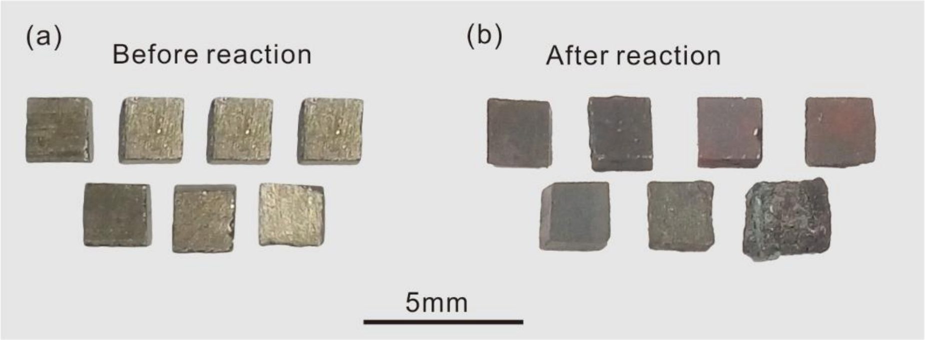

Fig. S3 Pyrite solids before and after reactions.

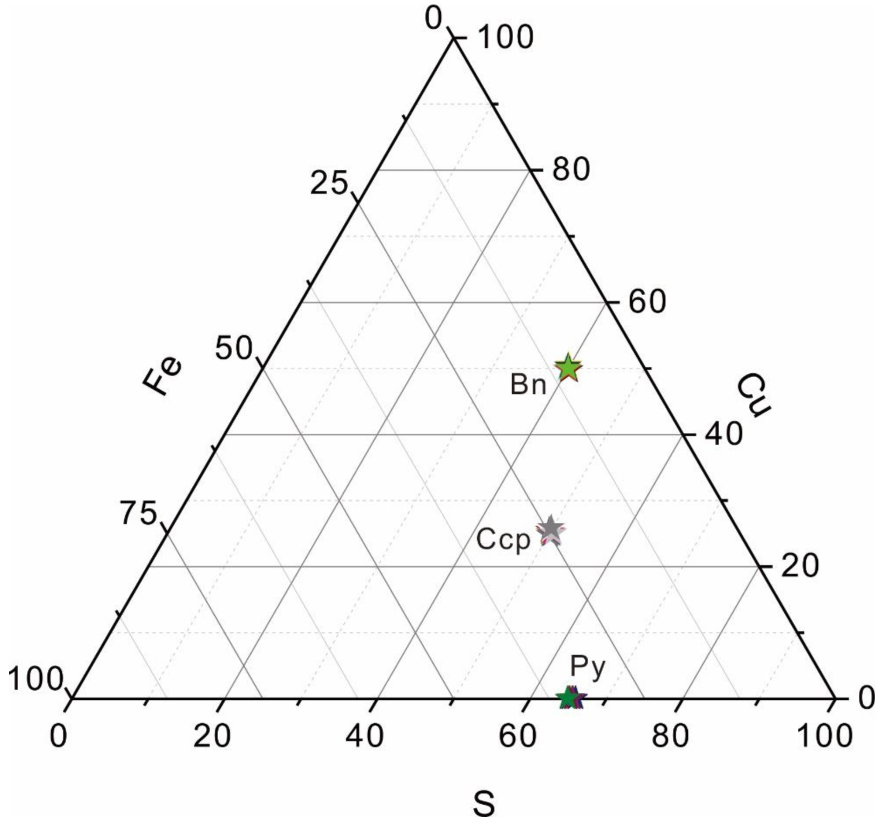

Fig. S4 Electron prob microanalysis results for pyrite/products (%). Py–pyrite; Ccp–chalcopyrite; Bn–bornite.

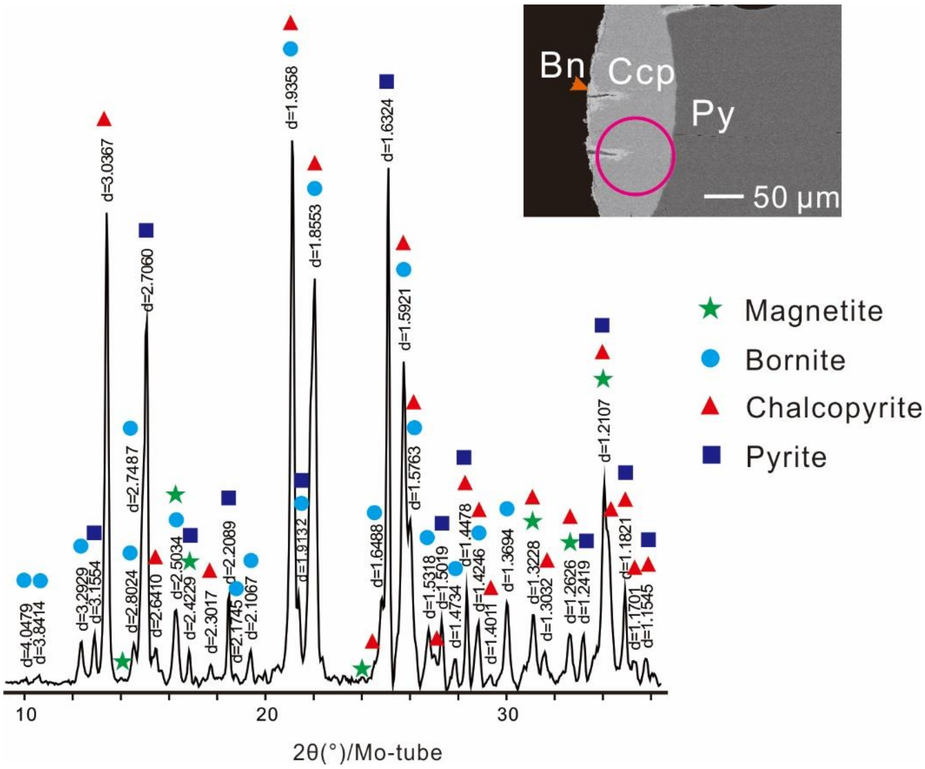

Fig. S5 Micro X-ray diffraction results demonstrating that the reaction products were chalcopyrite, bornite, and magnetite.

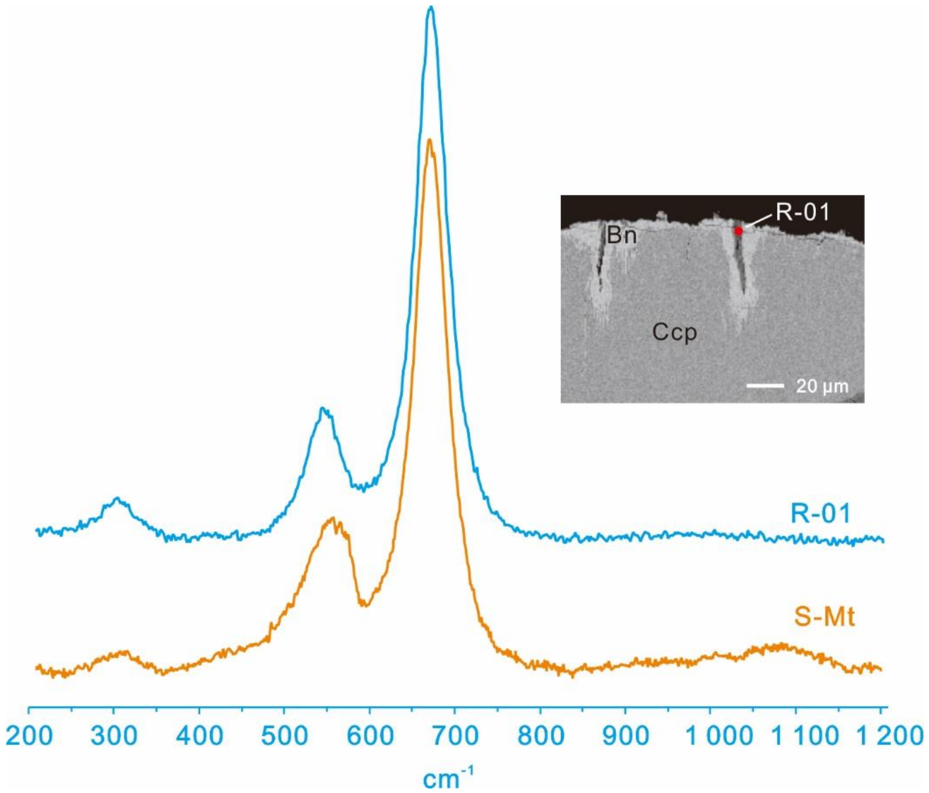

Fig. S6 Raman spectra showing the formation of magnetite. R-01 is the spectrum for the solid product in the reaction. S-Mt shows the standard spectrum for magnetite.

Table S1. Particles (pores) and cracks analysis system results for different types of edge lengths of pyrite grains

Run	Time (d)	Ltl [a] (μm)	Lunrl [b] (μm)	Lrl [c] (μm)
No.1	5	17852	8269	9583
No.2	11	23425	11853	11572
No.3	14	25474	12159	13314
No.4	21	27235	10033	17201
No.5	34	38302	15826	22475
No.6	48	43584	10704	32879
No.7	63	55855	5556	50298

Note: [a] Ltl- the total length of pyrite particle.

[b] Lunrl-the unrepleced length of pyrite particle.

[c] Lrl-the replaced length of pyrite particle.
